# Machine learning approaches for cardiovascular hypertension stage estimation using photoplethysmography and clinical features

**DOI:** 10.3389/fcvm.2023.1285066

**Published:** 2023-12-04

**Authors:** Saad Abdullah, Annica Kristoffersson

**Affiliations:** School of Innovation, Design and Engineering, Mälardalen University, Västerås, Sweden

**Keywords:** acceleration photoplethysmography, machine learning, cardiovascular, hypertension, photoplethysmography, clinical features, feature engineering

## Abstract

Cardiovascular diseases (CVDs) are a leading cause of death worldwide, with hypertension emerging as a significant risk factor. Early detection and treatment of hypertension can significantly reduce the risk of developing CVDs and related complications. This work proposes a novel approach employing features extracted from the acceleration photoplethysmography (APG) waveform, alongside clinical parameters, to estimate different stages of hypertension. The current study used a publicly available dataset and a novel feature extraction algorithm to extract APG waveform features. Three distinct supervised machine learning algorithms were employed in the classification task, namely: Decision Tree (DT), Linear Discriminant Analysis (LDA), and Linear Support Vector Machine (LSVM). Results indicate that the DT model achieved exceptional training accuracy of 100% during cross-validation and maintained a high accuracy of 96.87% on the test dataset. The LDA model demonstrated competitive performance, yielding 85.02% accuracy during cross-validation and 84.37% on the test dataset. Meanwhile, the LSVM model exhibited robust accuracy, achieving 88.77% during cross-validation and 93.75% on the test dataset. These findings underscore the potential of APG analysis as a valuable tool for clinicians in estimating hypertension stages, supporting the need for early detection and intervention. This investigation not only advances hypertension risk assessment but also advocates for enhanced cardiovascular healthcare outcomes.

## Introduction

Cardiovascular diseases (CVDs) are a leading cause of death globally, accounting for nearly 32% of all deaths in 2019 (1). Despite the impact of the COVID-19 pandemic, recent studies demonstrate that cardiovascular mortality remained the leading cause of death (2, 3). Notably, the World Health Organization (WHO) predicts that the mortality rate from CVDs will increase from 246 to 264 people per 100,000 during the period 2015–2030 (4–6). CVDs encompass a range of disorders that affect the heart and blood vessels, including coronary artery disease, heart failure, and stroke. Many risk factors have been identified for CVDs, including high blood pressure, high cholesterol levels, smoking, obesity, diabetes, and a family history of CVDs (7). Hypertension, or high blood pressure, is a particularly important risk factor for CVDs. It can lead to a number of complications for the heart, kidneys, and other vital organs, causing irreversible injury (6, 8). Therefore, early diagnosis, treatment, and control of hypertension could play a crucial role in the prevention and treatment of CVDs, whereas an effective management of hypertension can significantly reduce the risk of developing CVDs and other related complications (8).

**Figure 1:**
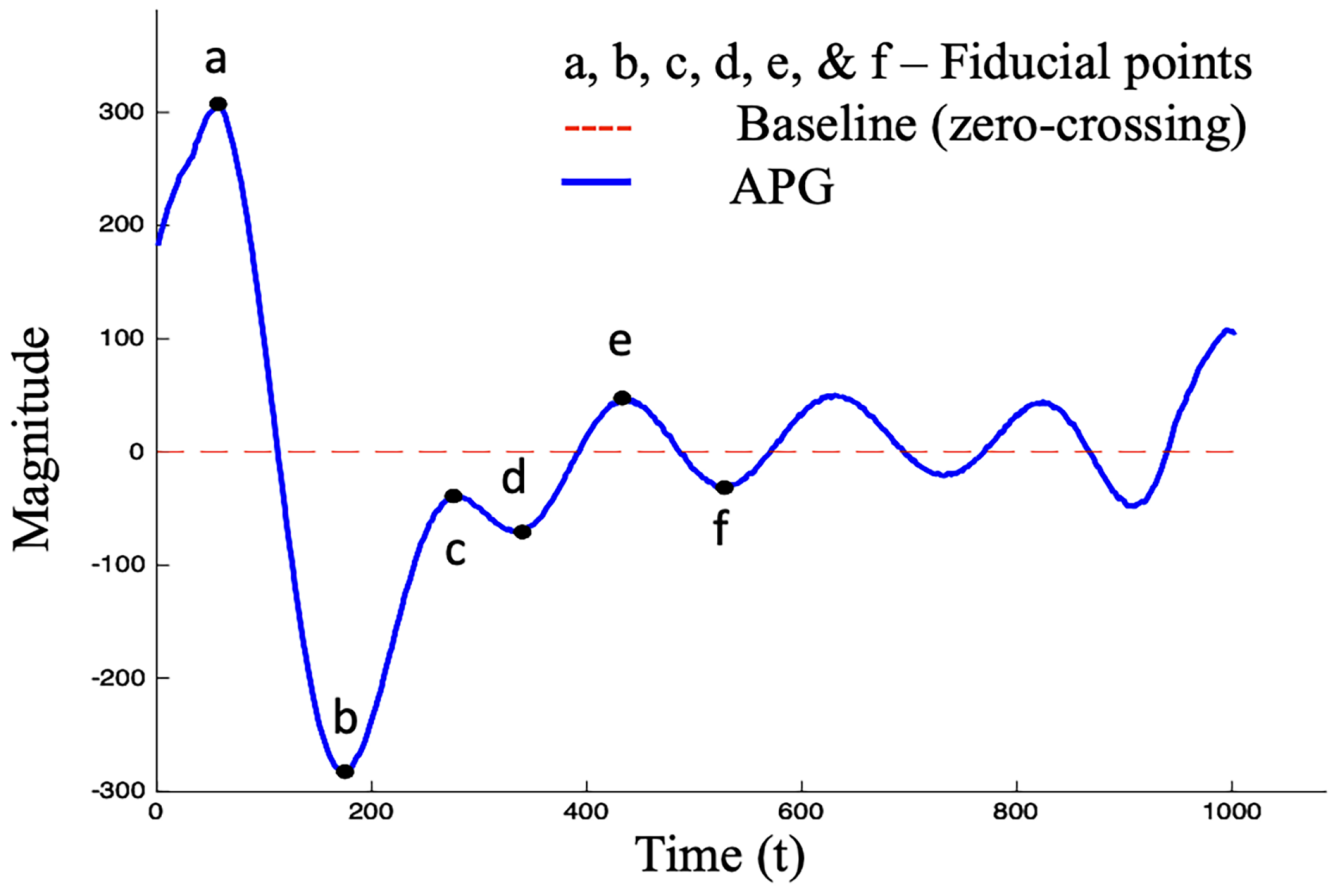
Standard APG waveform consisting of six fiducial points, where four points (**a–d**) are in the systolic phase and two points (**e, f**) are in the diastolic phase.

The analysis of Photoplethysmography (PPG) and its second derivative, namely Acceleration Photoplethysmogram (APG), has demonstrated significant potential for estimating blood pressure, particularly in patients with hypertension (9–11). Figure 1 shows the *c* and *d* fiducial points of the APG waveform which have been identified as sensitive indicators of CVDs (12). Numerous studies have demonstrated that the amplitude and timing of these points can provide valuable information about the aging process and support the assessment of cardiovascular disease (11, 13–17). The identification of the *c* and *d* points, however, is not trivial since they can be undetectable or non-prominent in the APG waveform (18, 19).

Artificial intelligence (AI) has ushered in unprecedented advancements in medical technology, pushing the boundaries of what was once unimaginable. AI is of relevance for precision medicine, natural language processing, expert systems, physical robots, patient engagement, and adherence applications (20). Even seemingly simple technologies like PPG have demonstrated remarkable success in establishing correlations with a diverse range of biological processes and systems particularly, through the computational capabilities of machine learning (ML) algorithms. This integration has significantly contributed to advancements in cardiovascular health assessment, respiratory monitoring, hemodynamic parameter estimation, autonomic nervous system evaluation, and the analysis of mental and emotional states, among others. By harnessing the potential of AI, healthcare professionals can now access a wealth of valuable information derived from PPG signals. The information enables more precise diagnoses, tailored treatments, and enhanced care of patients with CVDs (21).

## Background

Various studies have shown significant progress in this area, employing advanced techniques and achieving impressive results. Melin et al. (22) utilized neural networks and fuzzy inference systems to classify hypertension stages based on age, risk factors, and blood pressure pattern of behavior over a 24 h period, achieving a maximum classification performance of 98%. Singh et al. (23) demonstrated excellent results in diagnosing hypertension in patients with type I and type II diabetes mellitus, using a novel approach called rule extraction from support vector machines (SVMs). Das et al. (24) employed various modeling techniques such as Levenberg–Marquardt, gradient descent, and Bayesian resolution-based (BR-based) learning functions to model hypertension types in individuals aged 20–40. Shinde et al. (25) presented two distinct approaches for hypertension classification: information gain-based feature selection and genetic algorithm-based feature selection. These methods achieved classification accuracies of 97.58% and 99.19%, respectively. Abdullah et al. (26) presented a linear SVM model, employed to classify subjects as normal or at different stages of hypertension. The features used were a combination of statistical parameters derived from the APG *c* and *d* points and clinical parameters. The model achieved an overall accuracy of 87.50% on the validation dataset and a 95.35% accuracy on the test dataset.

Despite the progress made in hypertension detection and classification, alternative methodologies, and novel combinations of features to improve accuracy and early detection of cardiovascular diseases needs to be explored. In light of this, the current study focuses on a different approach, combining APG features and clinical parameters for hypertension classification using different ML algorithms.

## Method

In this study, we aim to develop a ML-based approach for estimating four different hypertension stages: (normal, prehypertension stage, stage 1 hypertension, stage 2 hypertension), using 19 features derived from statistical analysis of the APG waveform and the clinical parameters age, heart rate and systolic blood pressure. The features were extracted using the fiducial point extraction algorithms explained in our recently released MATLAB toolbox, PPGFeat (18).

We have used the publicly available PPG-BP Dataset (27), which contains three PPG segments collected with a sampling frequency of 1 kHz per subject, i.e., 2,100 sampling points. In the 3 min data collection phase, each PPG segment was assigned a Skewness SQI (Ssqi). The dataset includes data from 219 subjects, within the age range 21–86, and a nearly equal distribution of male and female subjects (48% and 52%, respectively). Out of 219 subjects, 80 subjects had normal heart conditions whereas 139 subjects had been classified as prehypertension stage, stage 1 hypertension or stage 2 hypertension.

**Figure 2:**
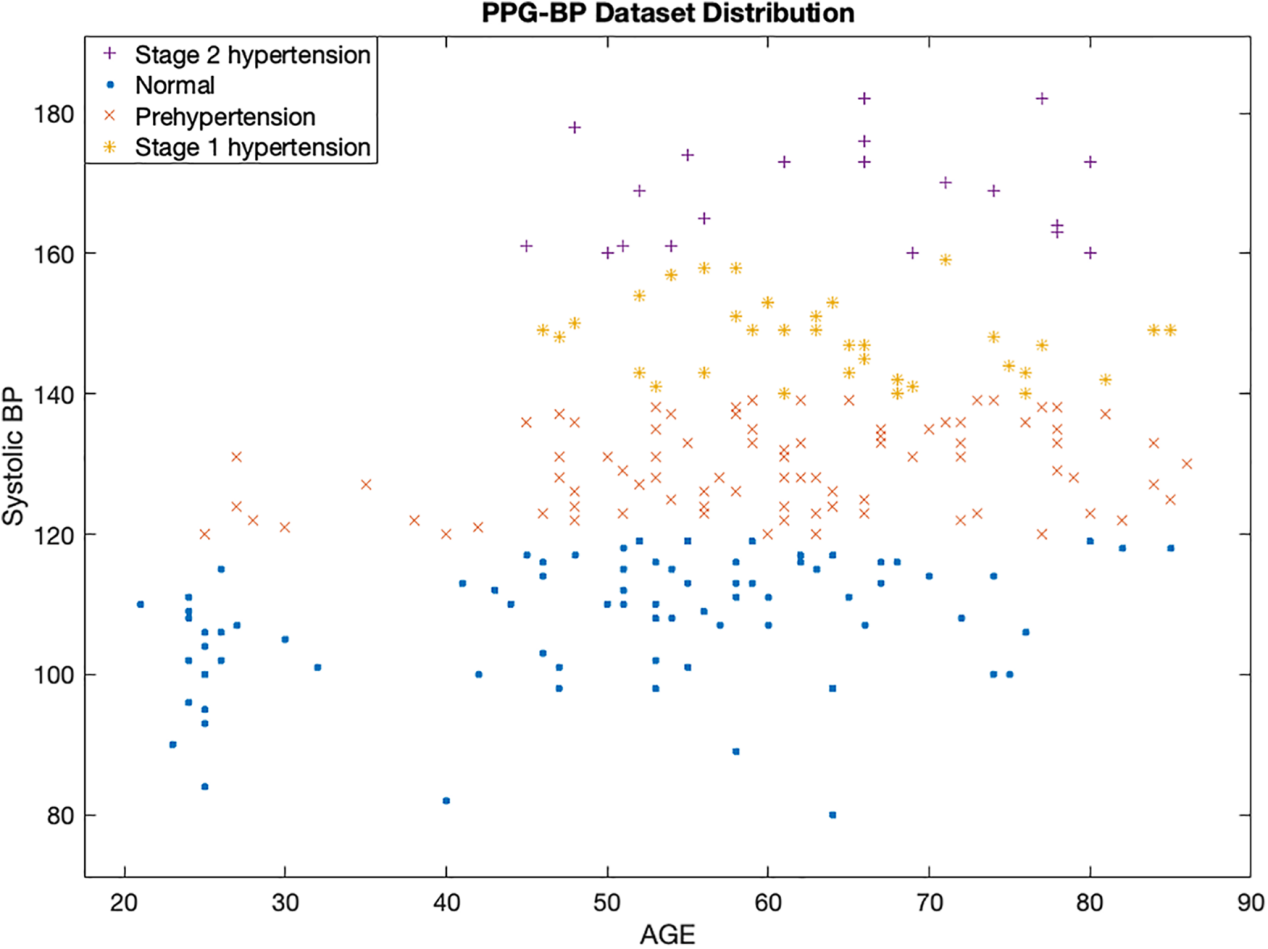
Scatter plot of PPG-BP dataset showing different classes of hypertension.

Figure 2 displays a scatterplot of the subjects at different hypertension stages in the PPG-BP dataset. It reveals an interesting insight into the relationship between age, systolic blood pressure, and hypertension stage. The *x*-axis represents the age of the subjects, while the *y*-axis represents their systolic blood pressure values. The different hypertensive stages are indicated by different colors of the data points. The scatter plot shows that there is a positive correlation between increased age and higher systolic blood pressure, which is associated with a greater risk of a CVD. However, it is also important to note that there are other factors beyond age, heart rate, and systolic blood pressure that influence hypertension stage.

By combining APG features and clinical parameters in our ML-models, we hope to improve the accuracy of CVD diagnosis and risk assessment. Our study will provide new insights into the use of APG waveform features and ML techniques for CVD diagnosis and contribute to the development of more effective and personalized approaches for cardiovascular healthcare.

### A machine learning algorithm for classification

In this study, we have proposed ML techniques to classify different stages of hypertension. We utilized three distinct algorithms to identify the most suitable approach for hypertension classification based on the dataset's multiple classes. The first algorithm utilized was the decision tree (DT) classifier, a powerful and interpretable model that makes decisions based on hierarchical structures of rules and thresholds (28). The DT utilizes the APG features and clinical parameters to recursively split the dataset into homogeneous subsets, leading to the creation of an efficient classification model.

Secondly, we applied linear discriminant analysis (LDA), a classic method widely used for supervised dimensionality reduction and linear classification tasks (29, 30). LDA aims to maximize the inter-class separation while minimizing the intra-class variability, thereby enhancing the discriminatory power of the features for hypertension classification. It exploits the linear relationship between features to find discriminant axes that best separate different classes, making it suitable for distinguishing between multiple hypertension types.

Lastly, we integrated the linear support vector machine (LSVM), a powerful classifier known for its effectiveness in high-dimensional spaces. LSVM seeks to find the hyperplane that best separates the different classes by maximizing the margin between support vectors (31). By incorporating the APG features and clinical parameters, LSVM optimizes the classification boundary, achieving accurate discrimination between hypertension stages.

### Preprocessing

**Figure 3:**
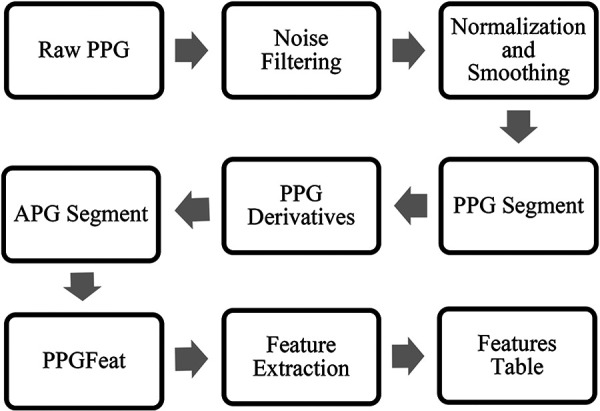
PPG waveform preprocessing steps, APG feature extraction and generation of a features table.

Figure 3 summarizes the various preprocessing stages performed on the raw PPG signals before the feature extraction, further information is presented in (19). These stages were necessary to remove noise and artifacts from the signal and enhance the quality of the data for accurate feature extraction.

The preprocessing stages included the filtering of the raw PPG waveform using a Chebyshev Type II, 4th order, 20 dB filter. Next, the data was normalized and smoothed using a moving average filter. Finally, the filtered PPG segment for each subject was obtained.

### Feature extraction

The study follows the APG fiducial point detection method proposed by Abdullah et al. (18, 19). Figure 3 summarizes the steps required to obtain features from APG. The algorithm takes a filtered PPG segment to obtain the APG, which is then processed through the PPGFeat (18) to extract relevant features from APG. In this study, we have focused on using the *c* and *d* points of the APG and performed statistical analysis on them for generating 16 time and amplitude features. The significance of these features has been discussed greatly in (32–34). In addition to the 16 statistical features, three clinical parameters were used as features, i.e., age, systolic blood pressure and heart rate. For ease in the ML-based classification, numerical classes were assigned to each subject, where class 0 was assigned to the normal subjects, class 1 was assigned to subjects with stage 1 hypertension, class 2 was assigned to stage 2 hypertension and class 3 was assigned to prehypertension stage.

### Features table

Table 1 provides a comprehensive list of the features derived from the APG waveform and utilized as input for the ML algorithms. The feature extraction process involved capturing the amplitude and time domain values of the *c* and *d* points. Furthermore, various other parameters, including the total time of the APG segment, were obtained to gain insights into the overall waveform characteristics. To enhance the feature set, a range of statistical features, such as distance, ratios, and slope, were then calculated from the extracted data (14, 35, 36). These carefully selected features collectively serve as critical inputs to the supervised ML algorithms, enabling accurate and reliable classification of hypertension stages.

**Table 1:** Features extracted from the APG waveform and the dataset used for the classification.

Statistical features from APG and clinical parameters			
1	$c_{t}$ (Time domain)	11	Slope of *c* and *d*
2	$d_{t}\;$ (Time domain)	12	*T* (total time of APG segment)
3	$c_{m}$ (magnitude)	13	$\frac{\mathrm{Distance}\;\mathrm{between}\;c\;\mathrm{and}\;d}{\frac{d_{t}}{c_{t}}}$
4	$d_{m}$ (magnitude)	14	$\frac{\mathrm{Distance}\;\mathrm{between}\;c\;\mathrm{and}\;d}{d_{m}-c_{m}}$
5	$d_{t}-c_{t}$	15	$\frac{\mathrm{Slope}\;\mathrm{of}\;c\;\mathrm{and}\;d}{T}$
6	$\frac{d_{t}}{c_{t}}$	16	$d_{t}-\frac{c_{t}}{T}$
7	$c_{t}-d_{t}$	17	*HR* (heart rate)
8	$\frac{c_{t}}{d_{t}}$	18	*AGE*
9	$\frac{c_{t}}{c_{m}}-\frac{d_{t}}{d_{m}}$	19	*SBP* (systolic blood pressure)
10	Distance between *c* and *d*		

## Results

Table 2 presents a comprehensive evaluation of three supervised ML-models: DT, LDA, and LSVM for CVD classification. The evaluation process involves two key steps: 5-fold cross-validation and testing. During cross-validation, each model is trained and tested five times on different subsets of the dataset to ensure robustness and reduce overfitting. The test dataset, consisting of 32 observations, is entirely separate from the training dataset, enabling an unbiased assessment of the models’ performance on previously unseen data.

**Table 2:** Performance comparison of ML-models on a multiclass classification**.**

Total Observations: 219		
Training Data Observations: 187		
Response Classes: 4		
Response Class Names: 0, 1, 2, 3		
Validation: 5-fold cross-validation		
Test Data Observations: 32		
Model Type	Accuracy % (Test)	Accuracy % (Validation)
DT	96.87	100
LDA	84.37	85.02
LSVM	93.75	88.77

The DT model (Table 2) demonstrates remarkable performance with an accuracy of 100% during cross-validation, indicating that it correctly classifies all instances in the validation data. During testing, the DT model achieves an accuracy of 96.87%, highlighting its ability to generalize well on new, unseen data. The model's hyperparameters were tuned using grid search optimization, with the split criterion set to Gini's diversity index (37). The grid search involved dividing the hyperparameter space into 10 grids, systematically exploring different combinations of hyperparameters to find the best configuration for optimal accuracy.

**Figure 4:**
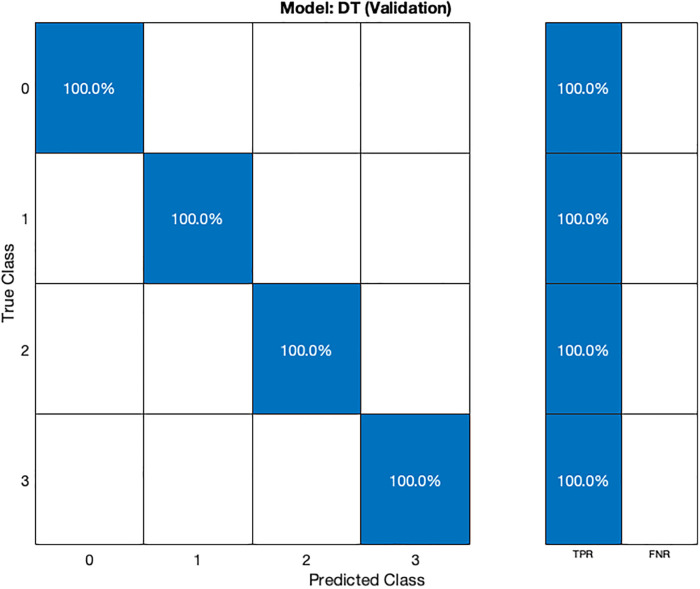
Validation confusion matrix for DT model.

**Figure 5:**
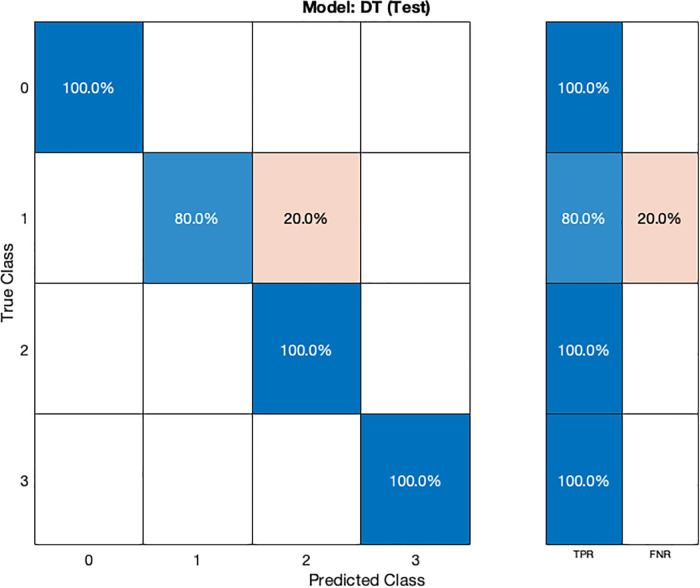
Test confusion matrix for DT model.

In the validation confusion matrix (Figure 4), the true positive rate (TPR) is 100% for all four classes, indicating that the model correctly identifies all instances of each class without any false negatives. In the test confusion matrix (Figure 5), the TPR for class 0, 2, and 3 remains 100%. However, for class 1, the TPR drops to 80%, implying that the model misclassifies 20% of stage 1 hypertension instances as other classes, leading to a 20% false negative rate (FNR) for class 1.

The LDA model (Table 2) exhibits competitive performance, achieving an accuracy of 85.02% during cross-validation and 84.37% on the test dataset. Hyperparameter optimization for the LDA model was carried out using Bayesian optimization, with 30 iterations to find the most favorable hyperparameter values for maximizing accuracy.

**Figure 6:**
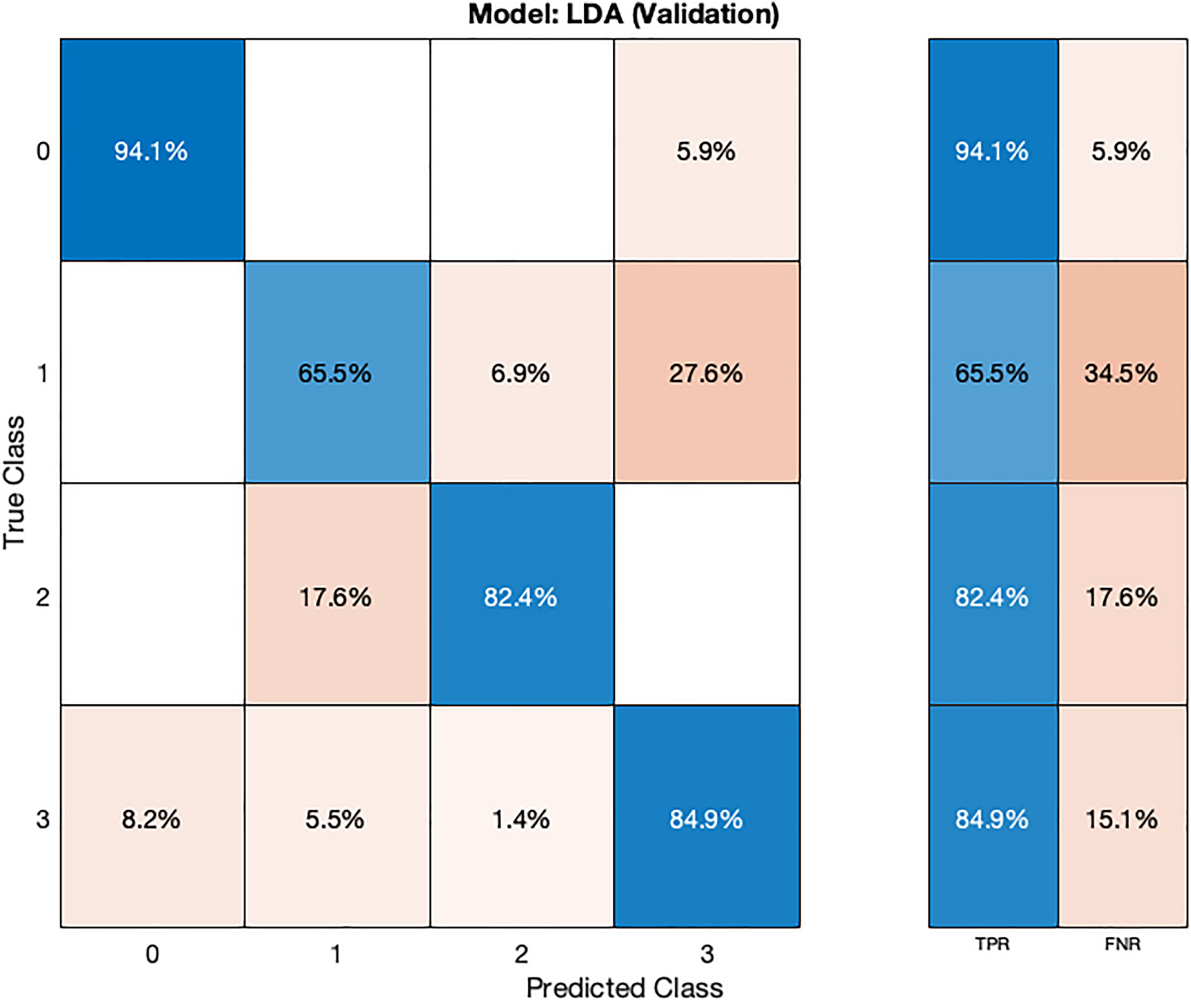
Validation confusion matrix for LDA ML model.

**Figure 7:**
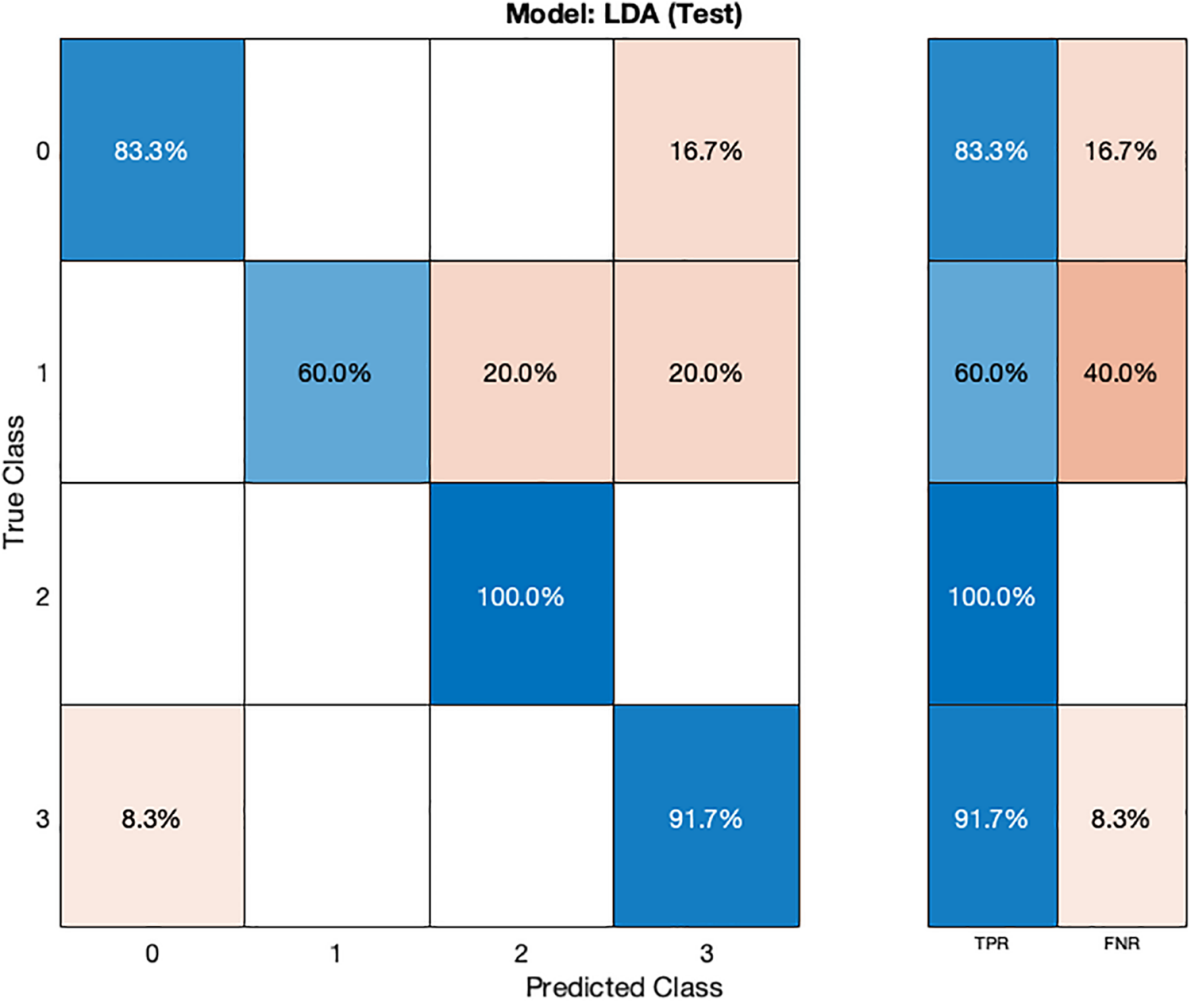
Test confusion matrix for LDA ML model.

In the validation confusion matrix (Figure 6), the LDA model achieves varying TPRs for each class: 94.1% for class 0, 65.5% for class 1, 82.4% for class 2, and 84.9% for class 3. The FNRs for the respective classes are 5.9%, 34.5%, 17.6%, and 15.1%. These results indicate the model's effectiveness in correctly identifying most normal subjects (class 0) but that the model is encountering challenges in distinguishing instances of stage 1 hypertension (class 1). In the test phase (Figure 7), the LDA model achieves a TPR of 100% for class 2 and 91.7% for class 3, indicating its ability to correctly identify all instances of these classes. However, it exhibits lower TPRs for class 0 (83.3%) and class 1 (60.4%), indicating difficulties in correctly classifying these instances. The corresponding FNR values for the test dataset are 16.7% for class 0, 40% for class 1, 0% for class 2, and 8.3% for class 3.

The LSVM model (Table 2) also demonstrates high accuracy, achieving 88.77% during cross-validation and 93.75% on the test dataset. Hyperparameter optimization for the LSVM model was performed using random search, involving 30 iterations to explore various hyperparameter combinations.

**Figure 8:**
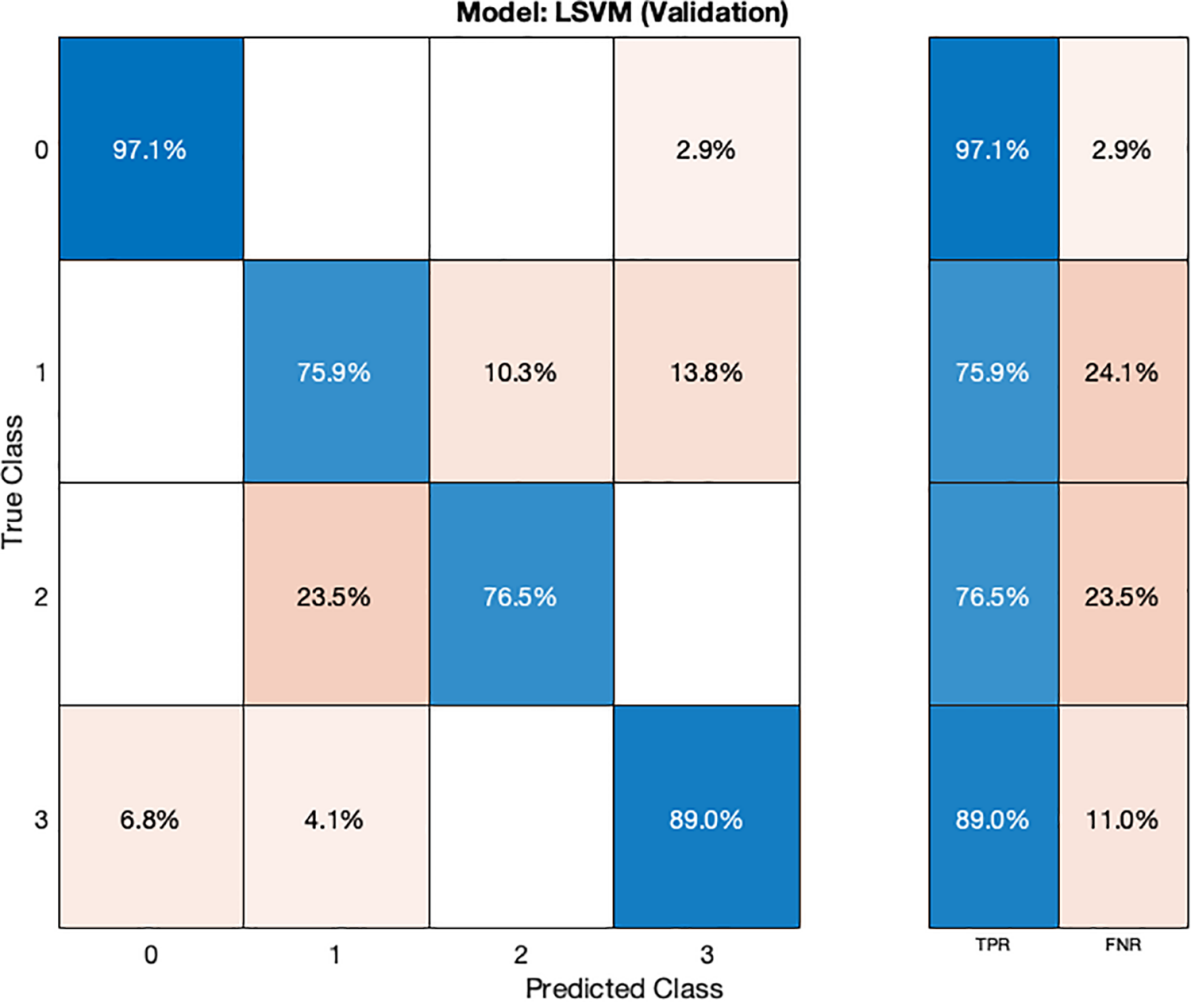
Validation confusion matrix for SVM ML model.

**Figure 9:**
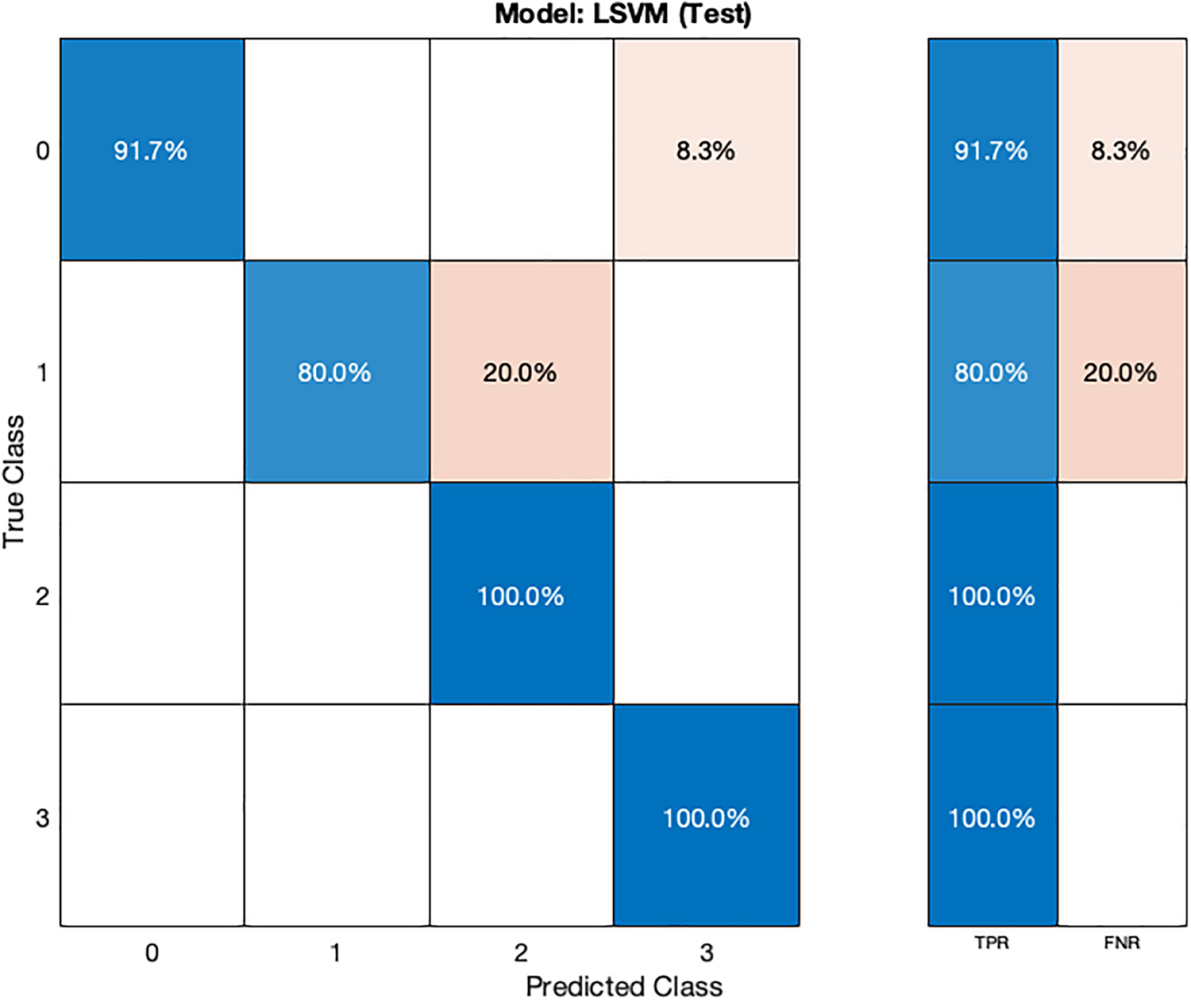
Test confusion matrix for SVM ML model.

In the validation confusion matrix (Figure 8), the LSVM model achieves TPRs of 97.1% for class 0, 75.9% for class 1, 76.5% for class 2, and 89% for class 3. These results indicate the model's ability to correctly identify a significant proportion of instances for each class during validation. However, it encounters more challenges in accurately classifying stage 1 hypertension subjects (class 1) as evidenced by a relatively lower TPR for this class. The corresponding FNRs are 2.9% for class 0, 24.1% for class 1, 23.5% for class 2, and 11.0% for class 3, reveals the specific types of misclassifications made by the model. In the test confusion matrix (Figure 9), the LSVM model exhibits high TPRs of 91.7% for class 0, 80% for class 1, and 100% for classes 2 and 3, demonstrating its accurate identification of instances in the testing dataset. However, the FNRs for class 0 and class 1 are 8.3% and 20%, respectively, indicating some misclassifications for these classes during testing. The LSVM model performs well in distinguishing stage 2 hypertension and prehypertension subjects, making it promising for hypertension classification.

The results show that the DT model achieves 96.87% accuracy on testing, demonstrating robust generalization, while LDA attains competitive results of 85.02% accuracy and LSVM achieves 93.75% accuracy. However, challenges arise in distinguishing stage 1 hypertension instances. Recommendations for future studies encompass refining models for better classifying stage 1 hypertension, expanding the dataset, incorporating additional clinical variables, and exploring model interpretability. This research advances hypertension risk assessment and highlights the potential for APG-based estimation in cardiovascular healthcare.

## Discussion and conclusion

The present study aimed to enhance hypertension diagnosis using ML by integrating APG features and clinical parameters. The research explored various ML-models and evaluated their performance in classifying normal subjects, and subjects with different stages of hypertension. The study showed that ML techniques have the potential to accurately classify different stages of hypertension. Three ML-models were employed for classification. The DT model exhibited exceptional performance, achieving a remarkable accuracy of 96.87% on the test dataset. It demonstrated robust generalization, correctly identifying instances from various classes. LDA and LSVM also exhibited competitive accuracies of 84.37% and 93.75%, respectively, showing their effectiveness in hypertension stages classification. However, all models faced challenges in accurately classifying instances of stage 1 hypertension, indicating a need for further improvement in this area.

PPG has been shown to be a useful tool for diagnosis and monitoring of various pathologies and physiological parameters and in recent years, it has been used with high accuracy as a substitute for BP and heart rate estimation (38, 39). Literature has shown that the PPG signal and the clinical parameters are valuable for diagnosing risk of cardiovascular diseases, especially hypertension (40, 41). The importance of preprocessing is evident in the quality of data used for feature extraction. To ensure accurate feature extraction, several preprocessing steps were undertaken. The raw PPG signals underwent Chebyshev filtering, normalization, and smoothing to remove noise and artifacts. The resulting filtered PPG segments provided a solid foundation for accurate APG features extraction. This process minimized the impact of noise and artifacts, ensuring that the extracted APG features were representative of cardiovascular health. Moreover, the APG features were extracted using the recently released MATLAB toolbox, PPGFeat (18). The toolbox's algorithms considered the preprocessed APG signals to accurately identify and extract relevant features, contributing to the comprehensive feature set used for ML classification.

The chosen features encompassed a range of statistical calculations derived from the *c* and *d* points of the APG waveform, capturing essential information about cardiovascular health. Additionally, the clinical parameters age, systolic blood pressure, and heart rate were included. These features collectively contributed to a comprehensive representation of physiological state, aiding accurate classification.

Hyperparameters played a crucial role in model performance. The DT model's hyperparameters were tuned using grid search optimization, resulting in exceptional accuracy. Similarly, Bayesian optimization was employed for hyperparameter optimization in LDA, while LSVM hyperparameters were fine-tuned through random search. The impact of hyperparameter tuning was evident in the performance of each model, underscoring the importance of selecting optimal values to enhance classification accuracy. The complexity of the ML- models varied. DT offers interpretability through hierarchical structures of rules, while LDA aims to maximize inter-class separation by reducing dimensionality. LSVM, known for its effectiveness in high-dimensional spaces, seeks optimal hyperplanes for classification. While all models demonstrated strong accuracy, the DT´s simplicity and interpretability stood out.

In comparison with previous studies, the current research presents a novel approach to hypertension classification by integrating APG features and clinical parameters with ML-algorithms. While previous studies have explored various techniques for diagnosing hypertension, such as neural networks, fuzzy inference systems, and support vector machines, the utilization of APG waveform features combined with ML algorithms represents a significant advancement in this field.

Melin et al. (22) employed neural networks and fuzzy inference systems to classify hypertension types based on age, risk factors, and blood pressure pattern of behavior over a 24 h period. Singh et al. (23) introduced rule extraction from support vector machines for diagnosing hypertension in patients with diabetes mellitus. Das et al. (24) used modeling techniques such as Levenberg–Marquardt and gradient descent to model hypertension types. Shinde et al. (25) presented information gain-based and genetic algorithm-based feature selection for hypertension classification. Abdullah et al. (26) utilized a linear SVM model with APG features and clinical parameters for hypertension classification.

While these previous studies have made notable contributions to hypertension diagnosis/classification, the current study differentiates itself by leveraging APG waveform features in combination with clinical parameters for ML-based classification. This approach enables a more comprehensive representation of cardiovascular health, capturing essential physiological information from the APG waveform. The integration of APG features enhances the discriminatory power of the classification models, leading to improved accuracy in identifying different stages of hypertension. Furthermore, the study's use of diverse ML algorithms, including DT, LDA, and LSVM, provides a comprehensive evaluation of classification performance.

Future endeavors will encompass the collection of diverse datasets and the investigation of APG features to enhance diagnostic precision. The study underscores the significance of the PPG-BP database as a pivotal resource for further research into PPG signals and cardiovascular health. As this research advances the comprehension of hypertension stages, future work could encompass the development of a real-time algorithm for detecting hypertension stages, cardiovascular health, and estimation of blood pressure, thereby promoting preventive strategies in cardiovascular healthcare.

## Limitations of the study

While this study makes a significant contribution in the realm of hypertension stage detection, it is important to acknowledge certain limitations and challenges that warrant consideration. One notable limitation lies in the inherent class imbalance within the dataset, which could potentially bias the performance of the developed ML-models. Furthermore, the reliance on a single dataset, the PPG-BP database, optimized for feature extraction using the PPGFeat toolbox, may introduce some level of dataset-specific bias and limit generalizability. The choice of clinical features and their associated threshold values, while based on established clinical significance, can also present potential limitations, especially when applied to diverse populations. Additionally, the interpretability of ML-models remains a challenge, especially in the context of medical decision-making. Finally, ensuring seamless integration of AI-driven diagnostic tools into clinical practice demands careful consideration of practical aspects, such as user interfaces and integration with electronic health records. By addressing these limitations and challenges, the field can advance toward more equitable, accurate, and clinically impactful AI-driven hypertension diagnosis and cardiovascular health assessment.

## Data Availability

Publicly available datasets were analyzed in this study. This data can be found here: https://doi.org/10.6084/m9.figshare.5459299.
